# Mechanical Pulling of Linked Ring Polymers: Elastic Response and Link Localisation

**DOI:** 10.3390/polym9080327

**Published:** 2017-08-01

**Authors:** Michele Caraglio, Cristian Micheletti, Enzo Orlandini

**Affiliations:** 1 Dipartimento di Fisica e Astronomia, Università di Padova and sezione CNISM, Via Marzolo 8, I-35131 Padova, Italy; michele.caraglio@pd.infn.it; 2 SISSA, International School for Advanced Studies, via Bonomea 265, I-34136 Trieste, Italy; michelet@sissa.it; 3 Dipartimento di Fisica e Astronomia, Università di Padova and sezione INFN, Via Marzolo 8, I-35131 Padova, Italy

**Keywords:** semiflexible rings, catenanes, topological links, linked portion, mechanical stretching

## Abstract

By using Langevin dynamics simulations, we study how semiflexible rings that are topologically linked respond to mechanical stretching. We use both constant-force and constant-velocity pulling protocols and map out how the mechanical tension affects observables related to metric quantities such as the longitudinal extension or span, and topology-related ones such as the length of the linked portion. We find that the average extension of linked rings, once divided by that of a single equivalent ring, is nonmonotonic in the applied force. We show that this remarkable feature becomes more prominent as the link complexity is increased, and originates from the different stretching compliance of the linked portion and the rest of the rings’ contour. By comparing the results of different pulling protocols, we also establish the best one for telling apart different types of links from their tensile response.

## 1. Introduction

Inter-chain entanglement can have major effects on the physical properties of polymer solutions or melts. An apt illustration is offered by biological systems, and particularly interphase chromosomes whose level of intermingling—Which could interfere with their capability to reorganize in later stages of the cell cycle—Is kept in check by active (enzymatic) and passive (physical constraints) mechanisms [1,2,3].

In systems where the free ends of the polymers can be chemically bridged, the aforementioned inter-chain entanglement can be trapped in the form of proper topological links [4]. Again, several examples are offered by biological systems. In bacteria, for instance, the replication of circular DNA produces daughter DNA rings that are interlinked and need to be separated by the action of topoisomerase enzymes. For the case of Trypanosoma parasites, instead, impressively extended chainmail networks of DNA rings known as kinetoplasts are actively maintained for functional reasons [5,6].

For these systems, as well as in the context of molecular nanomanipulation, a relevant question is whether and how the response to mechanical stretching depends on their linked state.

Besides the pratical implications, this problem is also conceptually appealing. In fact, it hinges on the interplay of topological constraints that act internally to the considered molecules and mechanical constraints that are imposed externally.

This interplay has long been studied both theoretically and experimentally for single polymer chains [7,8,9,10,11,12,13,14], DNA filaments [15,16,17,18,19] and knotted proteins tied in various knot types [20,21].

To our knowledge, the problem has remained practically unexplored for the case of linked rings or rings, except for a general overview presented in Ref. [22].

Here, we tackle it with a systematic study based on theory and numerical simulations of the stretching response of two identical semi-flexible rings that are topologically interlocked. In particular, we shall address the following questions: How does the stretching response of two linked rings compare to that of a single ring with the same total contour length? How do the force–extension profiles vary with link type? How does the size of the linked portion depend on the pulling load and link type?

To answer these questions, we carry out hundreds of stretching simulations using Langevin dynamics and compute conventional metric observables such as the projected distance of the anchoring points, as well as topology-oriented ones such as the length of the linked region.

Inspired by the typical setups of micromanipulation techniques based on atomic force microscopy (AFM) [23] or optical tweezers [24], we consider two different stretching protocols. In the first one, the two anchoring points (one on each ring) are pulled in opposite directions by a constant force (that is then varied), while in the second they are pulled away at constant speed.

The model, stretching protocols, and observables are introduced in Section 2, while the results are presented in Section 3. Section 4 is devoted to a discussion of the results and future perspectives.

## 2. Methods

### 2.1. Model and Simulation Setups

As model ring polymers, we consider semiflexible circular chains of N=120 beads of diameter σ. The potential energy of each ring includes three terms that describe the chain connectivity, excluded volume interactions, and the chain bending energy. The first two terms are, respectively, accounted for by a FENE potential [25] and a truncated and shifted Lennard-Jones potential,

(1) $$\begin{array}{ccc} U_{\mathrm{FENE}} & = & -\sum_{i}^{N-1}15\epsilon {\left(\frac{R_{0}}{\sigma }\right)}^{2}\ln\left[1-{\left(\frac{d_{i,i+1}}{R_{0}}\right)}^{2}\right]\;,\\ U_{\mathrm{LJ}}^{\mathrm{intra}} & = & \sum_{i,j>i}^{N}4\epsilon \;\left[{\left(\frac{\sigma }{d_{i,j}}\right)}^{12}-{\left(\frac{\sigma }{d_{i,j}}\right)}^{6}+\frac{1}{4}\right]\theta \left(2^{1/6}\sigma -d_{i,j}\right)\;\;. \end{array}$$

In these expressions, ϵ is the characteristic energy for the system, $d_{i,j}$ is the distance of beads *i* and *j*, $R_{0}=1.5\sigma$ is the maximum allowed bond length, and θ is the Heaviside function.

As it is customary in coarse-grained canonical simulations, we took the thermal energy of the system, $k_{B}T$, as the unit for measuring all energies, and set $\epsilon =k_{B}T$. Analogously, the bead size, σ, was taken as the unit of length. These choices are made to avoid committing a priori to a specific absolute value of temperature and lengthscales. These can in fact be conveniently mapped a posteriori based on the systems of interest.

The semiflexible character of the chain is instead accounted for by the following term:

(2) $$U_{\mathrm{bend}}=\sum_{i=2}^{N-1}K\left(1-\frac{{\vec{b}}_{i-1}\cdot {\vec{b}}_{i}}{\left|{\vec{b}}_{i-1}\right|\;\left|{\vec{b}}_{i}\right|}\right)\;,$$

where ${\vec{b}}_{i}\equiv {\vec{r}}_{i+1}-{\vec{r}}_{i}$ is the *i*th bond vector, with ${\vec{r}}_{i}$ being the position of the *i*-th bead. The bending rigidity parameter, *K*, is set to correspond to a nominal persistence length of $l_{p}=5\sigma$, implying that the chain will be rigid at scales smaller than 5σ and flexible at much larger contour distances.

**Figure 1:**
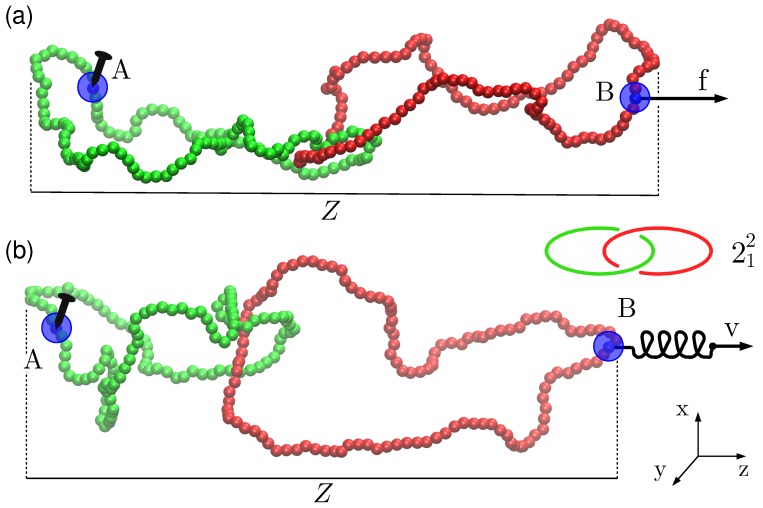
Schematic illustration of two semi-flexible rings topologically interlocked in a Hopf link and pulled with the (**a**) constant force and (**b**) constant velocity protocols. In both cases, bead A is a rooting point that is held fixed in space during the simulation, while bead B is pulled away from it along the *z* Cartesian axis. In the mode depicted in panel (**a**), a constant force is directly applied to bead B. In the alternative mode sketched in panel (**b**), bead B is attached to a harmonic spring whose other end is pulled away at constant velocity, *v*. The two examples clearly show that the anchoring points A and B—which initially correspond to the left-most and right-most beads—do not remain so throughout the simulated dynamical evolution.

The initial linked state of the rings is preserved at all times of the dynamical evolution by excluded volume effects. These steric interactions are enforced by a potential energy term, $U_{\mathrm{LJ}}^{\mathrm{inter}}$, which is analogous to $U_{\mathrm{LJ}}^{\mathrm{intra}}$ but acts on beads belonging to different rings. Finally, the mechanical stretching of the linked pair of rings along the *z* Cartesian direction is controlled via a pulling potential $U_{\mathrm{pull}}$ that takes on two different forms for the constant force and constant velocity protocols; see Figure 1.

The dynamical evolution of the system is described within a Langevin scheme. For the *i*-th bead, the stochastic equation of motion is

(3) $$m{\ddot{\vec{r}}}_{i}=-\xi {\dot{\vec{r}}}_{i}-\nabla U+\vec{\zeta },$$

where *m* is the bead’s mass, ξ is the friction coefficient, $\vec{\zeta }$ is the stochastic delta-correlated Gaussian noise, and $U=U_{\mathrm{FENE}}+U_{\mathrm{LJ}}^{\mathrm{intra}}+U_{\mathrm{bend}}+U_{\mathrm{LJ}}^{\mathrm{inter}}+U_{\mathrm{pull}}$ is the total potential energy of the system. Each Cartesian component of the noise has zero mean and variance equal to $\sigma_{\zeta }^{2}=2\xi k_{B}T$, as required by the fluctuation–dissipation relationship.

The equations of motion (3) are integrated numerically, starting from an initial configurations of the two rings that is prepared in one of several link types by a supervised procedure.

**Figure 2:**
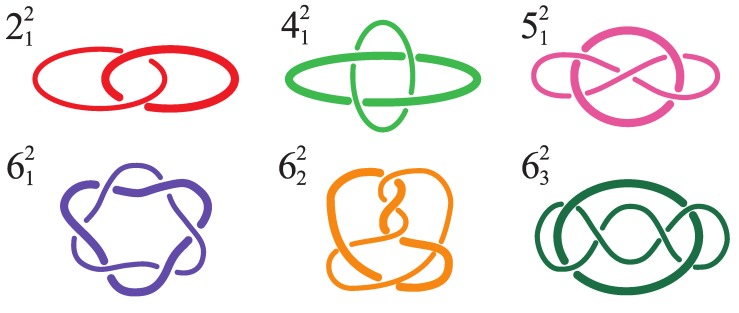
Sketches of two-component links considered in this study. For visual clarity, the components are drawn with different thickness. The links are listed by increasing complexity according to Rolfsen’s nomenclature [4]. The considered repertoire consists of: the simplest (Hopf) link type, $2_{1}^{2}$, the Solomon link, $4_{1}^{2}$, the Whitehead link, $5_{1}^{2}$, and the first three six-crossings links in the Rolfsen’s table, $6_{1}^{2}$, $6_{2}^{2}$, and $6_{3}^{2}$.

The link types considered here are sketched in Figure 2, where they are labelled according to the Rolfsen’s convention [4]. In Rolfsen’s notation, the primary number corresponds to total number of inter- plus intra-chain crossings in the minimal diagrammatic representation. This primary number is complemented with a superscript corresponding to the number of linked components, and a subscript which is a conventional index to distinguish different topologies with the same number of components and minimal crossings.

The repertoire of links considered here consists of two-component links only, and using Rolfsen’s notation, includes: the simplest (Hopf) link, $2_{1}^{2}$, the Solomon link, $4_{1}^{2}$, the Whitehead link, $5_{1}^{2}$, and the first three six-crossings links in the Rolfsen’s table, $6_{1}^{2}$, $6_{2}^{2}$, and $6_{3}^{2}$. Note that the Whitehead link is the simplest link with linking number equal to zero.

For each initial configuration, the integration is carried out with the LAMMPS simulation package with standard values for the friction coefficient and beads mass [25] and by using a time step $\Delta t=0.005\tau_{LJ}$, where $\tau_{LJ}=\sigma \sqrt{m/\epsilon }=\sigma \sqrt{m/k_{B}T}$ is the characteristic simulation time [26].

For each link type, we carry out 100 simulations where the system is first equilibrated for a total time span of $5\times 10^{5}\tau_{LJ}$, corresponding to $10^{7}$ integration steps, at zero load, $U_{\mathrm{pull}}=0$. The anchoring points for the pulling are set as follows. One anchor is identified with the bead with the lowest *z* coordinate. Next, on the partner ring, we pick as anchor the bead with the largest *z* coordinate. In the following, these two anchors are labelled as “A” and “B”, respectively (see Figure 1a). In both setups described below, anchoring point *A* is a rooting point held fixed in space, while point *B* is pulled away from it.

Note that because of the competition between configurational entropy and tensile energy, during the dynamical evolution of the system, the anchoring points A and B do not necessarily remain those with the smallest and largest *z* coordinates, respectively (nor is their distance vector guaranteed to remain parallel to the *z* axis). The actual rightmost and leftmost beads at any given time of the simulation are those actually used to define the longitudinal extension of the ring, Z, defined as the absolute difference of their *z* coordinates.

#### 2.1.1. Constant Force Setup

In the constant-force protocol, the pulling potential takes on the form

(4) $$U_{\mathrm{pull}}=-f\;\left(z_{B}-z_{A}\right)\;,$$

where $z_{A}$ and $z_{B}$ are the *z* components of the positions of anchoring points A (root, fixed in space) and B, respectively.

We considered 11 values of the force in the range $0\leq \tilde{f}\leq 32$, where $\tilde{f}$ is the adimensional, reduced force, $\tilde{f}\equiv \frac{f\sigma }{k_{B}T}$. We collect 100 trajectories at each force, starting from the largest value and reducing it to zero in a stepwise manner. At each value of $\tilde{f}$, we omit the initial timespan of $12\times 10^{7}$ integration steps when calculating the canonical averages for the observables of interest.

#### 2.1.2. Constant Velocity Setup

In the constant velocity protocol, the rooting point A is held fixed in space while the anchoring point B is progressively displaced along the *z* direction by the time-dependent potential

(5) $$U_{\mathrm{pull}}\left(t\right)=\frac{\lambda }{2}{\left(z_{B}\left(0\right)+v\;t-z_{B}\left(t\right)\right)}^{2}\;.$$

In this expression, $z_{B}\left(t\right)$ and $z_{B}\left(0\right)$ are, respectively, the *z* coordinate of point B at time *t* and at the beginning of the pulling action, t=0 (see Figure 1b).

For this protocol, we set the spring constant equal to $\lambda =\epsilon /\sigma^{2}$, the pulling velocity equal to $v=0.01\;\sigma /\tau_{LJ}$ and gather 100 independent trajectories, each of $2.5\times 10^{6}$ integration steps.

### 2.2. Locating the Linked Portion

#### 2.2.1. Top-Down Search

A key element of interest is how the applied tension affects the length of the region where inter-chain linking resides. Identifying this region—Which we term the *linked portion*—Presents conceptual challenges akin to those encountered for defining the knotted portion of entangled rings or open chains [27,28,29,30].

More precisely, to pinpoint the linked portion, one needs to overcome two different hurdles. The first is being able to define the linked state for two open curves (physical links). The second is to use the very same definition within a physically- and computationally-viable algorithm that locates the smallest portion of the two curves where the entanglement resides.

The former issue has been tackled by recent studies (all in protein-related contexts) that successfully dealt with open curves by using the Gauss linking number [31], by closing protein chains by bridging their termini on the surface of a distant sphere [32], via internal disulfide bridges [32,33], or even by using mechanical pulling protocols [34].

**Figure 3:**
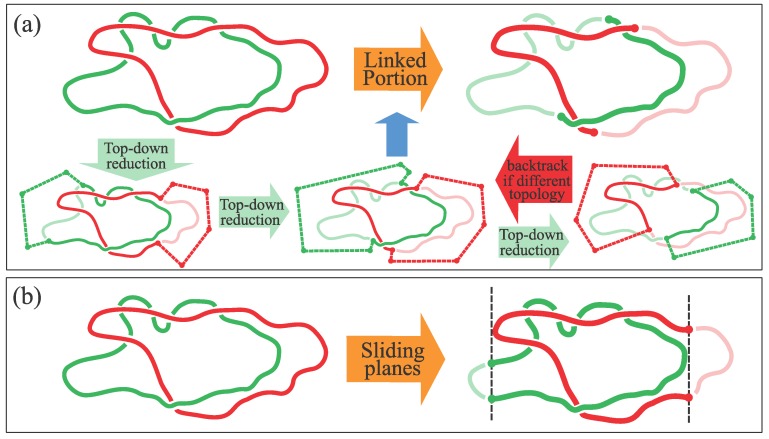
(**a**) Schematic illustration of the search strategy for locating the linked portion of two rings. The scheme is based on a top-down exploration of progressively shorter portions (arcs) of the two rings whose physically-linked state (established by closing each of them away from the center of mass of the partner arc) matches the initial linked state of the two rings. The search ends when the arcs cannot be further shortened without yielding a link type different from the original one. The viably-linked pair of arcs with the shortest total contour length, $\ell_{LK}$, is the sought linked portion. Adapted with permission from Ref. [22]. (**b**) Sketch of the “sliding planes" strategy for bracketing the linked portion. The dashed lines represent the projection of the planes (perpendicular to the pulling direction) that optimally bracket the linked portion. Each plane cannot be drawn closer to the other without intersecting both rings. The total contour length of the portion bracketed by the planes is indicated as $\ell_{LK}^{S}$.

The second challenge (i.e. locating the linked portion) was addressed in our recent study of Ref. [22]. The method—Which is adopted here as well—Consists of using a top-down search scheme to identify the shortest pair of subarcs from the two rings that—After suitable closure—Has the same linked state as the entire rings. The closure is carried out by extending the arcs’ termini “at infinity” in opposite directions with respect to the center of mass of the arc in the partner ring. This strategy is adopted to avoid as much as possible the formation of spurious inter-chain entanglement with the closure. A schematic illustration of the method is shown in Figure 3a. From now on, we denote by $\ell_{LK}$ the contour length of the (linked portion).

#### 2.2.2. Sliding Planes Method

To better illustrate the viability of the top-down approach, we shall contrast it with a simpler search strategy motivated by the following considerations of the strong stretching limit.

When the catenane is fully elongated, the linked portion is maximally tight (short) and located in the midpoint of the stretched catenane.

In this case, following the work of Ref. [11] on tensioned knotted chains, one can consider “slicing” the catenane with planes perpendicular to the pulling direction. Each of these planes will cut the link only in two points, except in correspondence of the linked portion. This is because in the high force limit, backfolds of the rings in the *z* directions are suppressed everywhere except in the region accommodating the essential crossings. One can hence bracket the linked portion by considering two perpendicular planes that slide inward from the anchoring points up to the point where further sliding would cut in more than two points. The region between the two planes cuts the catenane in two arcs that constitute—Within this second scheme—The linked portion (see Figure 3b).

The linked portions identified by the two methods, and their length, should be about identical in the limit of high stretching forces. As a matter of fact, the same search strategy has previously been successfully used to pinpoint the knotted portion in individual knotted chains subject to strong mechanical stretching. However, as one moves away from the limit of large pulling forces, backfolds in the *z* direction become more and more likely. In this case, perpendicular slices of the catenanes can cut through more than two strands, even far away from the genuine linked portion. At low or intermediate forces, this second method is hence expected to significantly overestimate the size of the linked region.

Accordingly, by examining the excess linked-portion length of the sliding plane method, one can expect to identify the onset of the strong stretching regime.

## 3. Results

### 3.1. Pulling at Constant Force

**Figure 4:**
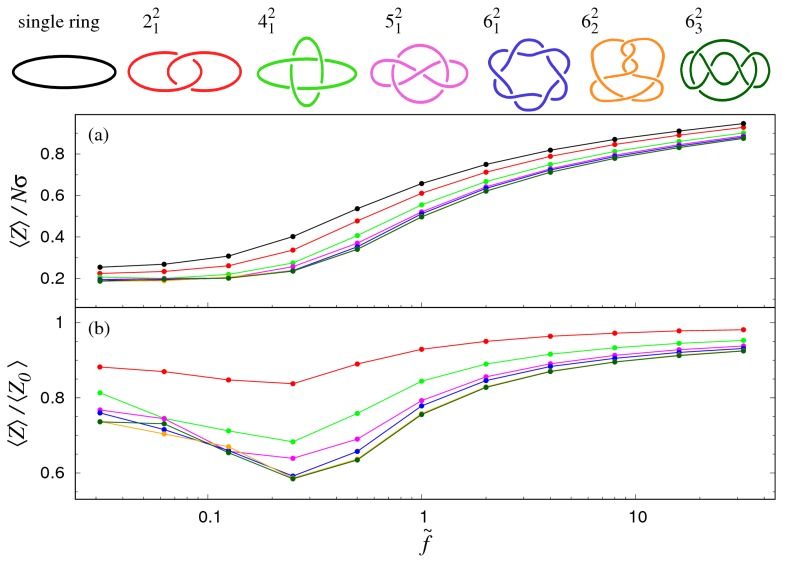
(**a**) Force–extension curves of pairs of rings interlocked in different types of topological links. Each ring consists of N=120 beads of nominal diameter σ. The average longitudinal extension along the pulling direction is indicated as 〈Z〉. In panel (**a**) the longitudinal extension is divided by Nσ, which is twice the maximum extension of an individual ring. In panel (**b**) the longitudinal extension of the link is divided by the average extension of a single ring of equivalent contour length, 2Nσ, at the same applied force.

We first consider how pairs of semiflexible rings which are topologically interlocked in various types of links respond to a constant stretching force. The results are shown in Figure 4, along with the stretching response of an unknotted ring of equivalent total contour length, 2Nσ.

For each link type, the force–extension curves in panel (a) have a sigmoidal trend, with an inflection point (at $\tilde{f}∼1$) marking the crossover between the weak and strong stretching regimes. At any given force, the extension of the single equally long ring is systematically larger than any other link type. This is clearly because part of the rings’ contour is topologically constrained (i.e., used up to maintain their specific linking state).

An interesting and unexpected feature is that this extension deficit varies non-monotonically with the applied force. This is more directly illustrated in Figure 4b, where at each value of $\tilde{f}$, the average extension of the link has been divided by $\langle Z_{0}\rangle$, which is the corresponding (force-dependent) extension of a single ring of equivalent length 2Nσ. One observes that the degree of non-monotonicity of the normalised force–extension curves increases with the link type, with a minimum at $\tilde{f}\approx 0.25$ for all considered topologies.

This intriguing property has a parallel with the stretching response of knotted chains [14], and can be ascribed to the presence of an additional lengthscale—The inter-chain entanglement length $\langle \ell_{LK}\rangle$—To the set of relevant lengthscales for the system metric properties.

**Figure 5:**
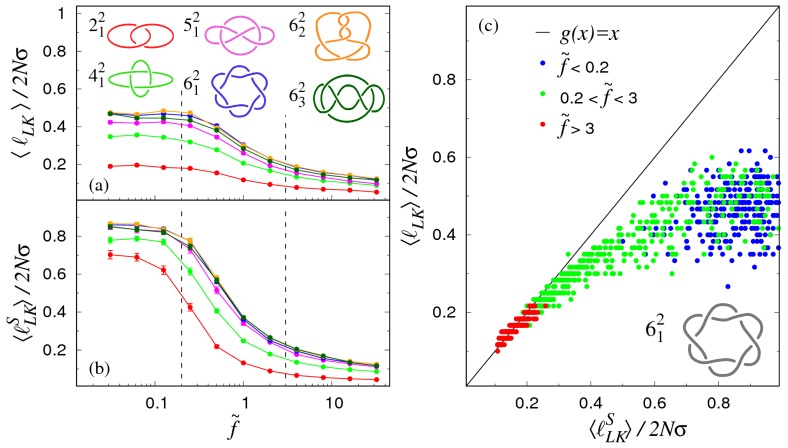
(**a**) Average contour length of the linked portion, $\langle \ell_{LK}\rangle$, as a function of the reduced pulling force $\tilde{f}=\frac{f\sigma }{k_{B}T}$. Different curves refer to different link types, as shown in the legend. The vertical lines are visual guides that approximately separate the weak $(\tilde{f}≲0.25),$ intermediate $(0.25≲\tilde{f}≲3),$ and strong $(\tilde{f}≳3),$ force regimes; (**b**) Same as in (**a**), but where $\langle \ell_{LK}^{S}\rangle$ from the sliding planes method, is used in place of the actual linked portion length; (**c**) Scatter plot of $\langle \ell_{LK}\rangle$ versus $\langle \ell_{LK}^{S}\rangle$ obtained for various linked configurations sampled at different values of the reduced force $\tilde{f}$ (see legend).

The dependence of $\langle \ell_{LK}\rangle$ on the applied tension is given in a semi-log plot of Figure 5a. The sigmoidal curve of each considered link type has its largest variation concentrated in the region $0.25≲\tilde{f}≲3.$ In particular, one notes that the weak force regime—Where $\langle \ell_{LK}\rangle$ deviates little from the $\tilde{f}=0$case—Holds up to $\tilde{f}∼0.25.$ This approximately coincides with the minimum of the rescaled longitudinal extension (see Figure 4b).

In fact, based on the data of Figure 5, one can put forward the following interpretation for the non-monotonicity of $\langle Z\rangle /\langle Z_{0}\rangle$.

For $\tilde{f}∼0.25$, the stretching response of a linked pair of rings is about equivalent to that of a ring with a definite but smaller effective length. This is because $\langle \ell_{Lk}\rangle$ remains about constant, implying that the contour length “stored” by the topological constraint is also constant. Dividing the catenane’s extension, 〈Z〉 by the one of the single ring, $\langle Z_{0}\rangle$, yields the initial decreasing trend of Figure 3b, because the ring is topologically-unconstrained (i.e., effectively longer, and hence with a higher stretching compliance).

At sufficiently high forces, the linked portion eventually tightens (see Figure 5), thus making more contour length available to the complementary topologically-unconstrained portion of the rings. This freed contour length then becomes available to the rings to further extend in response to the applied force. The extension gap with the single equivalent ring is therefore reduced systematically with increasing applied force. This second effect accounts for the increasing trend of $\langle Z\rangle /\langle Z_{0}\rangle$ for $\tilde{f}>0.25$.

We conclude the analysis of the linked portion length by comparing it with the estimate based on the sliding planes method sketched in Figure 5b.

The relationship between the two quantities is illustrated in the scatter plot of Figure 5c, where points are colored differently for low, mid, and high applied force (see legend). It is evident that the two measures are well consistent with each other in the strong stretching regime, $\tilde{f}>3$. Significant differences are instead noticed for $\tilde{f}<0.25$, where the sliding plane method returns a much larger contour length for the linked portion. This is clearly ascribable to the presence of backfolds along the *z* directions that become significant in this force regime.

### 3.2. Pulling at Constant Velocity

We conclude the analysis of the stretching response of linked semiflexible rings by considering an alternative pulling protocol. Specifically, we address the case in which the movable anchoring point B is pulled away from its initial position at constant velocity.

We set the velocity equal to $v=0.01\sigma /\tau_{LJ}$—A value set empirically so as to establish a competition between the time required to elongate the links and their intrinsic relaxation times. As we verified a posteriori (and discuss later), this velocity yields an appreciably different response with respect to the static (constant force) situation, and thus allows the out-of-equilibrium mechanical response of the system to be probed.

**Figure 6:**
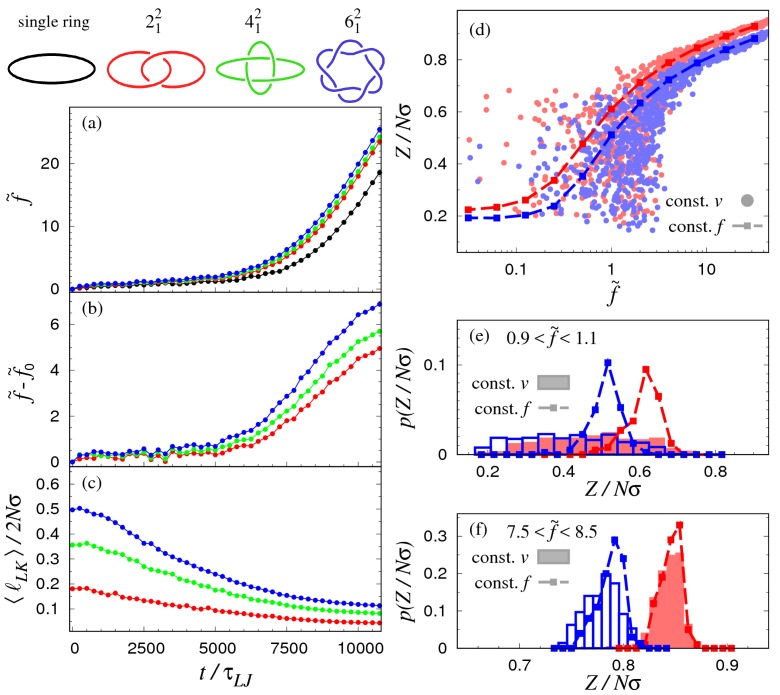
(**a**) Time dependence of the reduced force $\tilde{f}$ measured at the termini of various link types (see color-coded legend) pulled at constant velocity. The black curve is the time-dependent reduced force, ${\tilde{f}}_{0}$, for a single ring of equivalent length, 2Nσ, pulled with the same protocol. All curves are averages taken over 100 realizations. Panel (**b**) shows the difference of the time-dependent forces of the links and the equivalent ring; (**c**) Time dependence of the average contour length of the linked portion normalised to the total contour length of the link, 2Nσ; (**d**) Scatter plot of the extension, *Z*, and reduced force $\tilde{f}$ measured at equal times for the Hopf (red circles) and Star of David (blue circles) links. For comparison, the equilibrium force–extension curves (constant-force pulling protocols) are also provided. Normalised probability distributions of the normalised extension of Hopf and Star of David links for (**e**) large and (**f**) intermediate forces. The histograms pertain to the constant-velocity simulations, while the square datapoints and dashed lines outlines are for the constant-force protocol.

The time dependence of the force curves, averaged over 100 independent trajectories, are shown in Figure 6a for the single loop, the Hopf-link, the Solomon link, and the Star of David link.

One can visually identify two main dynamical regimes: for times smaller than $5\times 10^{3}\tau_{LJ}$, all curves are clustered together and show a weak time dependence. Beyond this timespan, the growth is steeper and clearly dependent on the link type. In particular, one notes that the largest forces are for the more complex links. The effect is more clearly illustrated in Figure 6b, which shows the force difference of each link type and the single equivalent ring.

The monotonic increase of force with link complexity at fixed time is intuitively explained with the longer “ropelength” [35] needed to tie more complex links. Clearly, increasing the topologically-constrained contour length leads to a decrease of compliance to stretching (i.e., a larger force).

In more quantitative terms, the effect is illustrated in Figure 6c, which shows what fraction of the total contour length of the catenane is occupied by the linked region at various pulling stages. For each considered link type, the curves decrease monotonically with time, and for a given time, the curves increase with the nominal complexity of the links.

The out-of-equilibrium character of the elastic response in Figure 6a is illustrated in the scatter plot of Figure 6d. The datapoints correspond to equal time values of the tensile force and the normalised extension for the Hopf and Star of David links. The points in saturated colors connected by dashed lines are instead the averages from the static constant-force simulations.

A noticeable qualitative difference between the two data sets is that the constant-velocity data points lie mostly below the constant-force curves, especially at low forces.

The effect is illustrated in panels (e) and (f) for datapoints picked in two different force ranges: $\tilde{f}\in \left[0.9,1.1\right]$ and $\tilde{f}\in \left[7.5,8.5\right]$. The distributions of the normalised elongation at these different forces are compared with constant-force data at $\tilde{f}=1.0$ and $\tilde{f}=8.0$, respectively. In the first case, shown in panel (e), one observes that the constant-force distributions for the Hopf and Star of David links are clearly separated, while in the constant-velocity case they overlap almost entirely. Additionally, the data from the latter case extend to far lower values of the extension with respect to the constant-force situation. At much larger forces (panel f), the constant-velocity and constant-force distributions are much more consistent, and both protocols allow for resolving the two link types. A systematic shift towards lower extensions is still noticeable for the constant-velocity stretching of the Star of David link.

## 4. Discussion

We have used Langevin dynamics simulations to study the mechanical stretching of a pair of topologically interlocked semiflexible rings. To our knowledge, a systematic analysis of such systems has not been carried out before, and therefore we addressed the problem by considering different types of links and pulling protocol (constant force and constant velocity) and by measuring various metric and topological observables. Besides the longitudinal span of the catenanes, these include the contour length of the linked portion; that is, the region of the linked rings where the topological entanglement is concentrated.

We find that the force–Extension curves of all link types, once normalised with respect to one of a single ring of equivalent contour length, are non-monotonic. Specifically, they feature a minimum when the reduced force, $\tilde{f}$, is about equal to 0.25.

This counter-intuitive non-monotonicity is reminiscent of the one found in knotted rings [14], and can be explained by considering the force dependence of the length of the linked portion. This is mostly constant and equal to its unperturbed value for $\tilde{f}≲0.25$ (small force regime). The contour length stored in this topologically-constrained region has a lower tensile compliance than the rest of the rings, and the extension falls progressively below the one of equally-long single ring as $\tilde{f}$ increases up to 0.25. Beyond this force, one enters the intermediate stretching regime, where the linked region starts to tighten. Now freed from the topological constraints of the linked portion, the released contour length can more effectively elongate in response to the applied force. The link extension now increases with $\tilde{f}$ more rapidly than the single equivalent ring. Finally, at $\tilde{f}∼10,$ one enters the strong stretching regime, where the linked portion is practically fully tightened for all link types.

The observed phenomenology indicates that it is the competition between the entropy of the contour length stored in the linked region and the tensile energy of the elongated linked pair that causes the intriguing non-monotonic behaviour of the force–extension curves and its dependence on the link topology.

The comparison of data from static constant-force simulations and the constant-velocity ones is finally used to characterise the out-of-equilibrium response of the linked pairs. Specifically, we observe that at intermediate forces, $\tilde{f}∼1$, the distributions of extensions from constant-velocities measurements are very broad and—Unlike the constant-force cases—Present substantial overlaps for different link types. At much higher forces, when the links are almost fully tightened, the extension distributions of the two protocols are in much better accord, and both techniques can resolve different link types, such as the Hopf and Star of David links.

These results can have valuable implications in the context of possible future force-spectroscopy experiments aimed at distinguishing different link types from the tensile response. We envisage that, for optimal resolution, such approaches should adopt a constant-force pulling protocol, or a constant velocity one where sufficiently high tensile forces are reached.

The present study could be extended in several directions. We believe it would be particularly interesting to analyse the tensile response of non-binary links, particularly multi-component catenanes and Brunnian links [4], which ought to yield an even richer phenomenology than observed here. A further interesting extension would be to elongate links not by applying mechanical tension, but by using spatial confinement, particularly narrow channels and slits [36], possibly also within a microfluidics setup.
